# Pralsetinib-associated pneumonia in RET fusion-positive non-small cell lung cancer

**DOI:** 10.1007/s00520-023-08125-3

**Published:** 2023-11-04

**Authors:** Ming Gao, Xia Zhang, Huan Yan, Decong Sun, Xuejiao Yang, Fang Yuan, Yanfang Ju, Lijie Wang, Jinliang Wang, Wei Zhao, Dong Zhang, Lin Li, Xiaoyun Xu, Junxun Ma, Yi Hu, Xiaotao Zhang

**Affiliations:** 1 Department of Oncology, The Fifth Medical Center of PLA General Hospital, No. 8 Fengtai East Street, Fengtai District, Beijing, 100071 China; 2 Department of Oncology, The First Medical Center of PLA General Hospital, Beijing, China; 3 Department of Oncology, The Second Medical Center of PLA General Hospital, Beijing, China; 4 Department of Oncology, Beijing Hospital, Beijing, China; 5 Department of Oncology, The First Central Hospital of Baoding, Baoding, Hebei China; 6 Department of Radiation Oncology, The Affiliated Qingdao Central Hospital of Qingdao University, Qingdao, China

**Keywords:** Pralsetinib, RET, Pneumonia, NSCLC

## Abstract

**Objective:**

Oncogenic alternation in RET is one of the important targets of non-small cell lung cancer (NSCLC). Pralsetinib has shown great efficacy in RET fusion-positive NSCLC, but a series of adverse reactions will inevitably occur in the meantime. We aimed to explore the clinical characteristics of patients with pneumonia and recognition it in early stage, so patients could longer benefit from pralsetinib.

**Methods:**

This is a multicenter, retrospective study. RET fusion-positive advanced NSCLC patients who developed pneumonia during pralsetinib treatment from January 2020 to December 2022 were included. Clinical data, time to onset of pneumonia, methods of pneumonia diagnosis, treatment with pneumonia, prognosis of pneumonia, and the effect of pneumonia on the efficacy of pralsetinib.

**Results:**

A total of 8 patients with pneumonia were included in the study, most of which were non-smoking female patients and the main fusion gene was KIF5B (87.5%), which was consistent with the general characteristics of RET fusion population. The median occurrence time of pralsetinib-associated pneumonia was 2.15 (range 1.1–6.63) months. All patients were infected by opportunistic pathogens, and the most common pathogen was human herpesviruses and pneumospora yerbii. Fever was always the first symptom, and timely anti-infective treatment including antibiotics, antiviral drugs, and antifungal drugs was effective. Until February 28, 2023, the median follow-up time was 18.7 months, the mean PFS of patients was 17.4 months, and the median PFS was not reached. Fortunately, patients who restarted pralsetinib after infection control continued to benefit.

**Conclusions:**

Opportunistic infection may be a unique adverse effect of pralsetinib. During the treatment of pralsetinib, we should be vigilant about the occurrence of pneumonia and achieve early recognition and timely treatment.

## Introduction

Lung cancer is one of the most common malignant tumors. The incidence of lung cancer ranks second only to breast cancer in the world, and the mortality rate ranks first [1]. Currently, molecular testing has become an important mean of detection in NSCLC. Common mutations include EGFR mutation, ALK fusion, ROS1 fusion, RET fusion, and MET exon 14 mutation [2].

RET gene is a proto-oncogene, located in the long arm of autosome 10, which encodes transmembrane receptor tyrosine kinase (RTK) and plays an important role in organogenesis and neural development during the embryonic period. RET binds to the ligand-receptor complex of the GDNF family to form a dimer complex that leads to autophosphorylation of intracellular tyrosine kinase domains and activation of downstream signaling pathways such as RAS/MAPK, ERK, PI3K/AKT, and JAK/STAT [3, 4]. The mutation of RET gene mainly generates fusion gene containing RET kinase domain and gain-of-function mutations in extracellular and cytoplasmic regions of RET protein through chromosome rearrangement, resulting in abnormal over-activation of independent ligands, thus promoting the occurrence of cancer. RET fusion accounts for about 1–2% of NSCLC, and the mutation rate is higher in younger age, female, and non-smoking population. The most common fusion genes are KIF5B (70–90%) and CCDC6 (10–25). Other fusion genes include NCOA4, TRIM33, ZNF477P, ERCC1, HTR4, and CLIP1 [4, 5].

With the development and marketing of selective RET inhibitors, selpercatinib and pralsetinib were successively approved by the FDA for the treatment of RET fusion-positive advanced NSCLC and thyroid cancer [6]. Pralsetinib is a small molecule RET inhibitor with high selectivity and affinity for RET. In vitro experiments, pralsetinib showed a 90 times higher selectivity for RET than VEGFR2, and its IC50 inhibitory effect on RET autophosphorylation was significantly lower than that of cabozantinib and vandetanib (5 nmol/L, 61.9 nmol/L and 833 nmol/L, respectively) [7].

A multi-cohort, open-label, phase I/II clinical study (ARROW) evaluated the efficacy and safety of pralsetinib in RET fusion positive NSCLC patients. The study showed that object response rate (ORR) of pralsetinib in the treatment of RET fusion positive NSCLC patients was up to 70% in treatment-naive group and 61% in previous-treated group. Median PFS was 9.1 and 17.1 months, demonstrating rapid and lasting clinical activity [8]. Therefore, pralsetinib was listed as the first choice of RET fusion positive first-line treatment in the NCCN guidelines.

The efficacy and safety of pralsetinib have been proven. Common grade 3 and above adverse reactions include hypertension, neutropenia, and anemia. Pralsetinib-associated pneumonia is a rare adverse event, accounting for about 15%. Clinically, pralsetinib-associated infectious pneumonia is easily confused with interstitial pneumonia and tumor progression. Therefore, this study collected patients who developed infectious pneumonia after pralsetinib. The characteristics of pneumonia and the possible mechanism were analyzed so as to improve the early identification and enable patients to benefit longer from pralsetinib.

## Method

### Patient selection

Patients with histologically or cytologically confirmed unresectable, locally advanced or metastatic NSCLC who developed pneumonia during pralsetinib were collected from January 2020 to December 2022. Molecular testing showed RET fusion positive, and patients receive pralsetinib (monotherapy or multi-agent therapy) as first or posterior line treatment. Pralsetinib-associated pneumonia was defined as pneumonia with imaging and etiological evidence after treatment with pralsetinib. Pneumonia with other clear causes of infection, immune checkpoint inhibitor–related pneumonitis, radiation pneumonitis, and interstitial pneumonia were excluded. Informed consents were obtained from patients enrolled in the study.

### Assessment

Basic information was collected including age, sex, tumor stage, mutation type, pathogenic bacteria, time to onset of pneumonia, methods of pneumonia diagnosis, treatment of pneumonia, prognosis of pneumonia, the effect of pneumonia on the efficacy of pralsetinib, and serial radiological imaging for pneumonitis with chest CT. Etiology can be diagnosed by culture, smear, serological experiment, polymerase chain reaction (PCR), and metagenomic NGS. Tumor evaluation was assessed by Response Evaluation Criteria for Solid Tumors (RECIST) 1.1. Adverse events were monitored according to Common Terminology Criteria for Adverse Events (CTCAE), version 5.0.

### Statistical analyses

Frequency analysis was used to describe the baseline information and characteristics of pneumonia.

## Results

We collected a total of eight patients who developed pneumonia after taking pralsetinib, all of whom were lung adenocarcinoma including 2 males and 6 females. The median age is 56 (range 34–81) years. One patient (12.5%) has a history of smoking, and two patients (25%) have brain metastases at initial diagnosis. The patients were in stage III (37.5%) or IV (62.5%), and the fusion genes include KIF5B (87.5%) and KIAA1468 (12.5%), among which 1 case was secondary RET mutation and the other 7 cases were primary mutation. 62.5% (5/8) of patients received pralsetinib as first-line treatment and 37.5% (3/8) as posterior line treatment (Table 1).

**Table1:** Characteristics of patients

Characteristic	Number (%)
Sex	
Male	2 (25)
Female	6 (75)
Median age (range), years	56 (34 ~ 81)
Smoking status	
Former or current	1 (12.5)
Never	7 (87.5)
Stage	
III	3 (37.5)
IV	5 (62.5)
Brain metastases	
Yes	2 (25)
No	6 (75)
Fusion gene	
KIF5B	7 (87.5)
KIAA1468	1 (12.5)
Lines of therapy	
First line	5 (62.5)
Posterior line	3 (37.5)

### Pneumonia

The clinical manifestations of pneumonia include fever, cough, phlegm, and chest tightness. Fever is the first symptom in 75% of patients. Pathogenic bacteria were detected mostly by sputum culture, bronchoscopic alveolar lavage fluid (BALF) NGS detection, and smear. One patient underwent magnetic navigation tracheoscopic biopsy, and fungal mycelia and spores were found pathologically. All the pathogenic bacteria were opportunistic, and the most common pathogen was human herpesviruses (62.5%), pneumospora yerbii (50%), followed by *cytomegalovirus* (37.5%) and *Aspergillus* (25%). (Table 2). A total of 75% of the patients were infected with multiple pathogens.

**Table 2:** Pathogen detection results and treatment

Patient	Sex	Age	Pathogenic bacterium	Detection method	Treatment
1	Male	55	Pneumospora yerbii, *Aspergillus fumigatus*, EBV	NGS, smear	Caspofungain, ganciclovir, meropenem
2	Female	34	CMV, EBV	NGS	Ganciclovir, moxifloxacin
3	Female	47	Pneumospora yerbii, HHV	NGS	Caspofungain, ganciclovir, moxifloxacin, SMZ-TMP
4	Female	44	Pneumospora, EBV	Serological experiment, sputum culture	Ceftazidime, moxifloxacin, SMZ-TMP
5	Female	64	CMV	PCR	Meropenem, ganciclovir
6	Male	81	*Cryptococcus*, *Staphylococcus aureus*	Smear	Voriconazole, fluconazole, vancomycin
7	Female	57	*Pseudomonas aeruginosa*, *Escherichia coli*, Haemophilus influenzae, *Aspergillus*, CMV, EBV, HHV	NGS	Voriconazole, ganciclovir, moxifloxacin, meropenem
8	Female	57	Pneumospora yerbii	NGS	SMZ-TMP

*EBV* Epstein-Barr virus, *CMV* cytomegalovirus, *HHV* Human herpes virus, *SMZ-TMP* paediatric compound sulfamethoxazole tablets, *NGS* next generation sequencing, *PCR* polymerase chain reaction

**Fig. 1:**
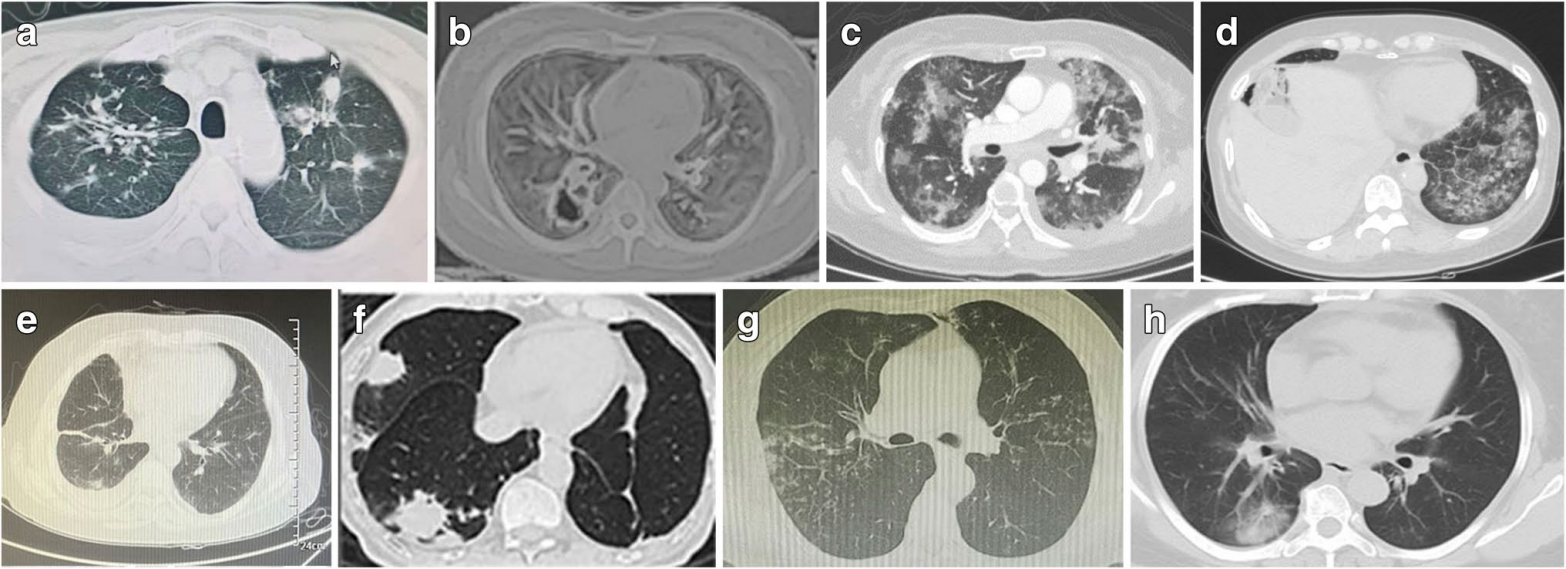
Imaging for pneumonia of patients 1–8 (**A**–**H**)

The imaging findings of CT scan can be single or multiple nodules, ground glass shadows, voids, or lung consolidation (Fig. 1). In case 1, the patient was mistaken for disease progression, but the effect of replacement therapy was poor, and he had recurrent fever. Laboratory examination showed an increased percentage of neutrophils, and NGS examination of bronchoalveolar lavage fluid (BALF) could detect fungi and viruses. The magnetic navigation tracheoscopy and biopsy were performed, and fungal infection of both pulmonary nodules was confirmed, so he was treated with antifungal drugs, and fever was relieved. He also continued to benefit from pralsetinib after retaking it.

Most patients developed infection within 6 months after pralsetinib, and the median time to pneumonia after application of pralsetinib is 2.15 (range 1.1–6.63) months. Patients were given anti-infective drugs including antibiotics, antiviral drugs, and antifungal drugs according to the pathogenic bacteria, and the median time to recovery from infection is 1.43 (0.60–4.30) months. One patient developed disease progression after infection control with an ECOG score of 4 and failed to continue treatment. The remaining patients resumed pralsetinib therapy and continued to benefit.

### Efficacy

**Fig. 2:**
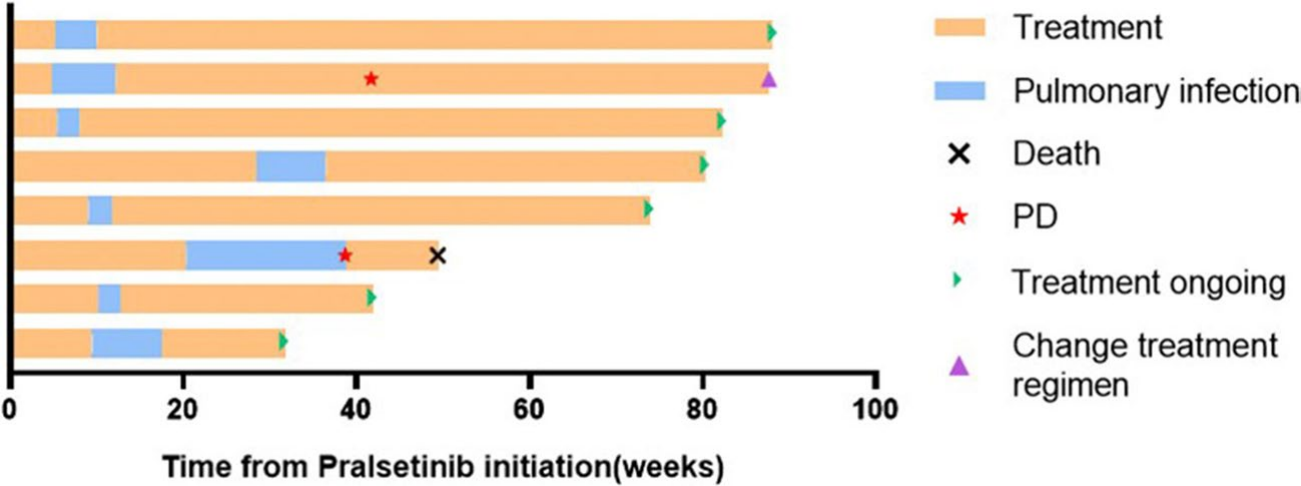
Treatment process of patients

According to RECIST 1.1 efficacy evaluation criteria for solid tumors, 5 patients achieved PR, and 3 patients were evaluated as SD, with an ORR of 62.5% in all and 80% in first-line treatment patients. Until February 28, 2023, the median follow-up time was 18.7 months. One patient died due to disease progression, 1 patient changed treatment regimens after progression, and 6 patients were still receiving treatment of pralsetinib (Fig. 2). The mean PFS of patients was 17.4 months, and the median PFS was not reached.

### Other treatment-related adverse events (TRAEs)

Other common TRAEs include leukopenia (50%), hypertension (37.5%), neutropenia (25%), and facial edema (25%), most of which were grade 1–2. One patient presented with grade 3 hypertension and was treated with pralsetinib reduction.

## Discussion

The incidence of pneumonia in patients treated with pralsetinib is relatively low, but if ignored, it can lead to serious complication and even life-threatening consequences. In ARROW study, 17% of patients developed pneumonia, and 8% are grade 3 or above events, including bacterial pneumonia, fungal pneumonia, viral pneumonia, and atypical pneumonia. In our study, 8 patients with pneumonia after pralsetinib were analyzed; all of them have lung adenocarcinoma, and most were female and non-smoking patients. The main fusion gene was KIF5B. The basic characteristics of the population were consistent with the characteristics of the total population with RET fusion. Among the 8 patients included in the study, the pathogens included fungi, bacteria, and viruses, with the common characteristic of all opportunistic pathogens. Pralsetinib was restarted in 7 patients after the infection controlled. The median follow-up time was 17.4 months, and the median PFS was not reached, indicating that timely and effective treatment of pneumonia does not affect the efficacy of pralsetinib.

Opportunistic infections caused by pralsetinib have also been reported abroad. Rashmi Dhital et al. in the USA reported a case of spirillum infection in a patient who took pralsetinib [9]. Another Korean study reported two cases of pralsetinib-associated extrapulmonary tuberculosis [10], suggesting that opportunistic infection may be a unique adverse effect of pralsetinib.

The cause of pneumonia induced by pralsetinib is currently unknown and may be related to the downstream pathway of RET gene. Abnormal fusion of RET can activate RAS/MAPK, ERK, PI3K/AKT, JAK/STAT, and other downstream signaling pathways. Pralsetinib is a receptor tyrosine kinase RET inhibitor, which can selectively inhibit RET kinase activity and then inhibit the phosphorylation of RET and its downstream molecules in a dose-dependent manner. Studies have shown that inhibition of PI3K pathway can decrease the number and function of regulatory T cells and inhibit the inflammatory response of NK cells and neutrophils. JAK/STAT signals are involved in the regulation of CD4 + T cells. JAK kinase inhibitors can inhibit the activation and differentiation of CD4 + T cells [11]; reduce the number of Th1, Th17, and regulatory T cells (Tregs); inhibit the activation and maturation of NK cells; and easily cause immunosuppression, thus increasing the risk of infection [12]. Therefore, pralsetinib may affect cellular immunity by inhibiting the RET downstream pathway and the function of T cells and NK cells, leading to increased risk of infection, especially opportunistic infection.

Pathogens that cause opportunistic infections include bacteria, fungi, viruses, and protozoa. Most opportunistic fungal infections are caused by Candida and Aspergillus [13]. Our study shows that the common pathogen of infections is Pneumocystis yerinii. Pneumocystis pneumonia is the most common cause of pneumonia in immunosuppressed patients, especially in AIDS patients. Pneumocystis pneumonia is mainly caused by decreased CD4 + T cell count or functional deficiency, which further proves that the mechanism of pralsetinib-induced infection may be related to cellular immunity.

Pneumonia can appear about 2 months after taking pralsetinib, and its early diagnosis and timely treatment are particularly important. The diagnosis is based on laboratory, etiological, and imaging examination. In case of unexplained fever, the infection indicators and chest CT should be examined immediately. Once pneumonia is clear, if there is no exogenous infection factor, it can be considered as pralsetinib-related infection. Then, oral pralsetinib should be suspended, or the dosage should be reduced under close monitoring. Initial empirical therapy with antimicrobial agents should be initiated as soon as possible before microbiological findings are detected, given the possibility of immunosuppression in the patient. If patients have a history of steroid use, they should be alert to the possibility of opportunistic infections, especially fungal infections, and antifungal drugs can be used.

Pralsetinib-associated pneumonia should be distinguished from tumor progression and interstitial pneumonia. The imaging findings of fungal infection may be nodular, solid, or ground glass, and some may form irregular voids. Nodular lesions can easily be confused with tumor lesions. A retrospective study analyzed the occurrence and nature of invasive pulmonary mycosis in patients with NSCLC, and among the 13 patients confirmed with pulmonary mycosis, 62% were initially mistaken for metastatic or recurrent lung cancer [14], indicating the importance of differential diagnosis between the two.

Interstitial pneumonia is a common adverse effect of targeted therapy. In EGFR-TKI, afatinib developed interstitial pneumonia in 10% of patients and oxitinib in 4% [15]. Chest CT scan of interstitial pneumonia can present as diffuse ground glass, grid-like shadows, or honeycomb shadows in both lungs. Normally, no clear pathogen can be identified for interstitial pneumonia. Patients with interstitial pneumonia usually respond to steroid and are not sensitive to anti-infective drugs. If a patient is misdiagnosed with interstitial pneumonia and given hormone shock therapy, the use of large amounts of hormones may aggravate the fungal infection and eventually progress to severe pneumonia. Therefore, early and continuous detection of NGS has guiding significance for the discovery and treatment of pneumonia.

## Conclusion

The incidence of pralsetinib-associated pulmonary infection is relatively low, but it can cause serious consequences without timely diagnosis and treatment. The detection of pathogenic bacteria of NGS is of guiding significance for early diagnosis. Opportunistic infection may be a unique adverse reaction of pralsetinib, and early detection and timely application of anti-infective drugs are the key to treatment. At present, the specific mechanism of pralsetinib-associated opportunistic infection is not clear and needs further exploration.

## Data Availability

The data of this study are available on request from the corresponding author upon reasonable request.
